# Quantification of Niacin and Its Metabolite Nicotinuric Acid in Human Plasma by LC-MS/MS: Application to a Clinical Trial of a Fixed Dose Combination Tablet of Niacin Extended-Release/Simvastatin (500 mg/10 mg) in Healthy Chinese Volunteers

**DOI:** 10.1155/2015/212437

**Published:** 2015-08-05

**Authors:** Pingping Zhang, Yantong Sun, Guobing Shi, Yin Sui, Qiuying Li, Yunbiao Tang, Jingkai Gu

**Affiliations:** ^1^Department of Pharmacy, General Hospital of Shenyang Military Area Command, No. 83, Wenhua Road, Shenhe District, Shenyang 110840, China; ^2^Research Center for Drug Metabolism, School of Life Sciences, Jilin University, Changchun 130012, China

## Abstract

Our paper aimed to develop rapid, sensitive, and specific LC-MS/MS method for the quantification of niacin (NA) and its metabolite nicotinuric acid (NUA) in human plasma. Following protein precipitation with acetonitrile, the NA, NUA, and internal standard (5-fluorouracil) were separated on a Zorbax 300SB-C_8_ column (250 mm × 4.6 mm, 5 *μ*m) with a mobile phase consisting of methanol-2 mM ammonium acetate (3 : 97, v/v) at a flow rate of 1 mL/min (split 1 : 1). A tandem mass spectrometer equipped with electrospray ionization source was used as the detector and operated in negative ion mode. The linear concentration ranges of the calibration curves were 5–800 ng/mL for NA and NUA. The intra-assay RSD for quality control (QC) samples were from 5.0% to 8.7% for NA, and 5.5% to 7.6% for NUA. The interassay RSD for QC samples were from 2.8% to 9.4% for NA, and 3.7% to 5.8% for NUA. The relative errors for QC samples were from −2.2% to 2.3% for NA, and −0.6% to 3.2% for NUA. The method was successfully applied to the investigation of the pharmacokinetic profiles of NA, NUA in human after single dose administration of Niacin extended-release/Simvastatin tablet (500 mg/10 mg).

## 1. Introduction

Atherogenic dyslipidemia is highly prevalent, especially in patients with insulin resistance and diabetes mellitus. Atherogenic dyslipidemia increases the risk for coronary heart disease and peripheral vascular disease and remains a serious public health problem [1]. Niacin (nicotinic acid, 3-pyridine-carboxylic acid) (NA) is a water soluble vitamin and belongs to the vitamin B complex [2]. It possesses the ability to decrease low density lipoprotein (LDL) cholesterol level and to increase high density lipoprotein (HDL) cholesterol level which led to its clinical use in the treatment of dyslipidemia and prevention of atherosclerosis [3]. Simvastatin (SV) belonging to the class of pharmaceuticals called statins is a specific and nonreversible competitive inhibitor of HMG-CoA reductase, which is used for lowering cholesterol in the patients with hypercholesterolemia and for preventing cardiovascular disease [4, 5]. It was found that a statin combined with NA can be an attractive option because both have excellent records of improving cardiovascular outcomes and can effectively correct all abnormalities of atherogenic dyslipidemia in patients especially those with diabetes [6]. According to the US Food and Drug Administration (FDA) guidelines, a fixed dose combination of NA and SV for use in patients with complex lipid abnormalities has been approved in 2008 [4]. It may lead to the attainment of lipid regulation goals when monotherapy with SV or NA is considered inadequate. Furthermore, the combination of two lipid-lowering agents in one formulation may potentially improve patient compliance. A product containing extended-release NA (dose range from 125 to 1000 mg per tablet) and SV (dose range from 5 to 40 mg per tablet) obtained from literature was under extensive investigation and conclusions were drown that SV plus NA regimen was effective, safe, and well tolerated in patients with or without diabetes mellitus [4, 5, 7–9]. NA was rapidly metabolized in vivo to two main metabolites through two pathways: the first is the metabolic route to nicotinuric acid (NUA) and the second is that to Niacin amide (NAM). NAM is further transformed to nicotinamide, nicotinamide-N-oxide, and two pyridone derivatives [10]. The first pathway was thought to be the mechanism responsible for the flushing side effect and hepatotoxicity of NA in human. Moreover, the concentration of NUA was higher than NAM; therefore NUA was selected as an estimation goal [10–12].

Various methods are available for determination of NA alone or NA along with its metabolites in biological fluids, like determination of NA and its metabolites by LC-MS/MS following deproteinization with acetonitrile in rat or human [3, 12–14] or with methanol in human or dog [5, 15], liquid-liquid extraction with ethyl acetate in human [10, 16, 17], and also solid phase extraction in human [2]. And also there are some investigations about NA combined with statins drug formulations in animal or humans [5, 11, 16, 17]. Based on literature survey, we developed a specific LC-MS/MS method for the quantification simultaneously of NA and NUA in human plasma after single dose oral administration of a pharmaceutical formulation containing Simvastatin 10 mg and Niacin 500 mg to healthy Chinese volunteers. By doing this, we hope this study can be of help in substantiating the clinical use of Niacin extended-release/Simvastatin tablet in the treatment of human atherogenic dyslipidemia.

## 2. Experimental

### 2.1. Chemicals and Standards

NA, NUA, and 5-FU were purchased from National Institute for the Control of Pharmaceutical and Biological Products (Beijing, China). The purities of these compounds were found higher than 98%. Methanol and acetonitrile of HPLC grade were purchased from Fischer Scientific (Fair Lawn, NJ, USA). All other chemicals and solvents were of analytical grade. Milli-Q water used throughout the study was obtained from a Millipore system. Drug-free human plasma was provided by the General Hospital of Shenyang Military Area Command.

### 2.2. Instruments

#### 2.2.1. Mass Spectrometry

The mass spectrometer was API 4000 triple quadrupole system equipped with a TurboIonSpray ESI interface operated in negative multiple reaction monitoring (MRM) mode to monitor the transitions *m*/*z* 122.0→*m/z* 78.0,* m/z* 178.7→*m/z* 78.0, and* m/z* 128.9→*m/z* 42.1 for NA, NUA, and 5-FU, respectively. The optimized value of declustering potential (DP) and collision energy (CE) were −30 V and −17 eV for NA, −40 V and −25 eV for NUA, −48 V and −26 eV for 5-FU. Data acquisition was carried out with Analyst 1.5.1 software (AB MDS Sciex).

#### 2.2.2. Liquid Chromatography

Agilent 1100 system (Palo Alto, CA, USA) was used for solvent and sample delivery. The chromatographic separation for two analytes and IS was achieved by using a Zorbax 300SB-C_8_ column (250 mm × 4.6 mm, 5 *μ*m; Agilent, USA) protected by C_8_ guard column (4.0 × 3.0 mm, 5 *μ*m; Phenomenex, Torrance, CA, USA). The mobile phase was comprised of methanol-2 mM ammonium acetate (3 : 97, v/v) at a flow rate of 1 mL/min (split 1 : 1). The total LC analysis time per injection was 4.5 min with isocratic elution. An injection volume of 40 *μ*L was used for all samples.

### 2.3. Preparation of Standard and Quality Control Samples

Two separate stock solutions of the analytes were prepared at 1 mg/mL in methanol-water (50 : 50, v/v) and stored at −20°C. A series of standard working solutions for NA and NUA were prepared by diluting stock solution with methanol-water (50 : 50, v/v). The 5-FU solution was brought to a final concentration of 1000 ng/mL in acetonitrile from 1 mg/mL in acetonitrile. All working solutions were stored at 4°C and brought to room temperature before use. Calibration standards and quality control samples were prepared by spiking 50 *μ*L of the working solutions and 50 *μ*L of IS into 100 *μ*L of drug-free human plasma. Matrix-matched NA and NUA calibration standards were prepared in plasma at concentrations of 5, 10, 20, 60, 200, 600, and 800 ng/mL, respectively. Similarly, NA and NUA quality control (QC) samples were prepared separately in plasma to give concentrations of 10, 60, and 600 ng/mL.

### 2.4. Sample Preparation

Extraction of NA and NUA was carried out by protein precipitation. First, 50 *μ*L of the IS working solution (1000 ng/mL 5-FU) and 50 *μ*L of water were added to a 100 *μ*L aliquot of human plasma. The sample mixture was then deproteinized with 250 *μ*L of acetonitrile, and the precipitate was removed by centrifugation at 15000 rpm for 10 min. The supernatant was transferred to another tube and evaporated under a gentle stream of nitrogen at 40°C until it is dry. The residue was reconstituted in 150 *μ*L mobile phase and vortex-mixed for 30 sec. An aliquot of 40 *μ*L of the solution was injected into the LC-MS/MS system for analysis.

### 2.5. Method Validation

Measurements for each analyte in the biological matrix should be validated according to relevant guidelines [18, 19]. Method development and validation included (1) selectivity; (2) accuracy and precision; (3) recovery and matrix effect; (4) the calibration curve; (5) lower limit of quantification; (6) dilution integrity; and (7) stability of analytes in spiked samples.

Selectivity is performed by using blank samples from at least six sources. Peak areas of endogenous compounds coeluting with the analytes should be less than 20% of the peak area of the LLOQ standard.

The lower limit of quantification (LLOQ) is considered being the lowest calibration standard and established using at least five samples. The calibration curves were defined in three separate runs on the basis of duplicate assays of the spiked plasma samples, and, on the same day, QC samples from three concentrations (Table 1) were determined in replicates (*n* = 6) to calculate the method's accuracy and precision. Calibration curves were constructed using a 1/*x* ^2^ weighting linear regression of the peak-area ratios of the analyte to the IS versus the plasma concentration of the analyte.

Extraction recovery was measured at three levels by comparing the response of the analyte spiked before and after sample preparation. Matrix effects were assessed using a method similar to that reported by Matuszewski et al. (see below) [20].

Dilution integrity was evaluated by spiking the plasma with an analyte concentration above ULOQ and diluting this sample with blank plasma when processing. We investigated three concentrations by dilution of 20 times of QC samples.

Stability studies of NA and NUA should investigate the different storage conditions over time periods that equal or exceed those applied to the actual study samples. The following stability tests were evaluated: (1) stability of the stock solutions of the analytes; (2) freeze and thaw stability of the analytes in the plasma from −20°C to room temperature for three cycles; (3) short-term stability of the analytes in plasma at room temperature before extraction for 3 h and the ready-to-inject samples (after extraction, in the mobile phase) to the autosampler rack for 4 h; and (4) long-term stability of the analytes in matrix stored at −20°C for two months. All stability tests samples at three concentration levels (10, 60, and 600 ng/mL) were analyzed in triplicate and the deviations were determined in relation to freshly prepared samples.

### 2.6. Application of the Method

The validated method was applied to the quantification of NA and its metabolite NUA in human plasma obtained from a clinical trial. It was in accordance with the Helsinki Declaration of 1975 (revised in 2008) [21] and approved by the institutional ethics committee.

Before conducting the study all subjects provided written informed consent to participate. The study was designed as randomized, open-label, crossover, and single dose periods with a 7-day washout between each treatment. A total of 12 healthy Chinese volunteers (6 males and 6 females) aged 20 to 35 years with a body mass index between 19 and 24 kg/m^2^ were recruited. Subjects were randomized into three groups (2 males and 2 females for each treatment). They were orally dosed with Niacin extended-release/Simvastatin 1, 2, and 3 tablets once daily, separately. The venous blood samples were collected at time intervals (0, 0.33, 0.66, 1.0, 1.5, 2.0, 2.5, 3.0, 3.5, 4.0, 5.0, 6.0, 8.0, 12, and 24 h) in separate vacutainers during the treatment. All blood samples were centrifuged at 3000 ×g for 10 min and the plasma was separated and kept frozen at −20°C until analysis.

### 2.7. Data Analysis

The pharmacokinetic parameters were calculated with data analysis system (DAS) program (version 2.0, Mathematical Pharmacology Professional Committee, Shanghai, China). The maximum plasma concentration (*C*_max_) and the corresponding time (*T*_max_) were obtained from observed data. The terminal elimination rate constant (*K*_*e*_) was estimated with least-squares regression of values in the terminal log-linear region of plasma concentration-time curves, and the terminal elimination half-life (*t*_1/2_) was calculated to be 0.693/*K*_*e*_. The area under the curve from time zero to the last sampling time (AUC_0−*t*_) was determined using the linear trapezoidal rule.

Statistical analysis of pharmacokinetic parameters was performed using SAS version 9.1 (SAS Institute Inc., Cary, North Carolina, USA). A *P* value of <0.05 was considered statistically significant.

## 3. Results and Discussion

### 3.1. LC-MS/MS Optimization

**Figure 1:**
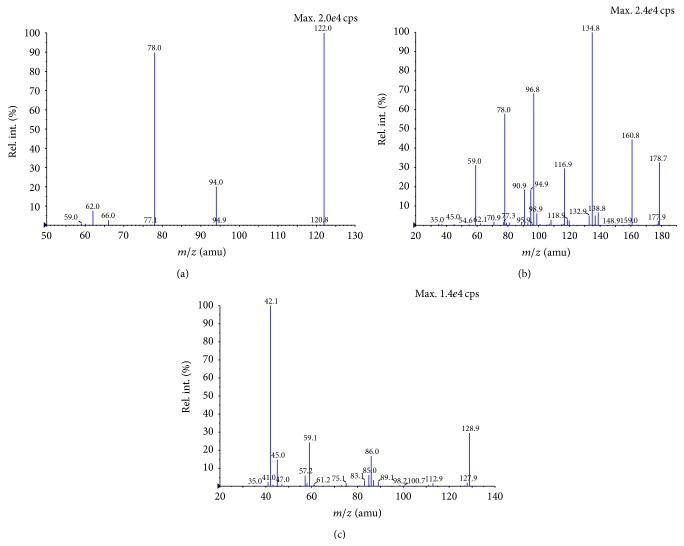
Product ion mass spectra of the deprotonated molecules of NA (a), NUA (b), and 5-FU (c).

We used ESI-MS/MS to analyze the compounds. To quantify the analytes using the MRM mode, the full scan and product ion spectra of the analytes and IS were investigated. Owing to the similar functional group pyridine ring and carboxyl group of NA and NUA, it makes them give excellent sensitivity in the negative ESI mode. The deprotonated peak was the predominant form of the molecular ion for NA at* m/z* 122.0 and NUA at* m/z* 178.7. Because of the similar pyridine parent ring, the dominant fragments observed for NA and NUA were both at* m/z* 78 (Figure 1). The fragment ion* m/z* 78, formed by breaking the single carbon-carbon bond between the pyridine ring C-2 site and side chain carbonyl group, was present in the greatest abundance and hence was selected as the product ion for quantitation. Figure 1 displays product ion spectra of the deprotonated molecules of NA, NUA, and their corresponding IS.

In order to optimize the LC system for the detection of NA and NUA, chromatographic separation was tested on several C_18_ and C_8_ columns to achieve the best efficiency and peak shape: Phenomenex Gemini (150 mm × 2.0 mm, 5 *μ*m), Capcell Pak MG (150 mm × 2.0 mm, 5 *μ*m), and Discovery (50 mm × 2.1 mm, 5 *μ*m), while the asymmetrical peak was acquired on the Zorbax 300SB-C_8_ column (250 mm × 4.6 mm, 5 *μ*m; Agilent, USA) for NA and NUA because of good peak shape and satisfactory recovery. Different mobile phases were evaluated to improve LC separation and enhance MS sensitivity. Methanol was selected as the organic modifier because its ability to ionize NA and NUA was superior to that of acetonitrile. Moreover, the addition of ammonium acetate (2 mM) in the mobile phase greatly increased signal intensity for NA and NUA. Thus, an isocratic system with a mobile phase consisting of methanol and 2 mM ammonium acetate (3 : 97, v/v) was optimal for NA and NUA with respect to peak shape and mass spectral response. Under these chromatographic conditions, the retention time for NA and NUA was approximately 4.5 min.

### 3.2. Internal Standard Selection

We have chosen 5-FU as an internal standard. Although a stable isotope-labeled compound would be an ideal IS for quantitation in complex matrices in LC-MS/MS analysis, it is not readily available in most laboratories. Hence, structural analogues were screened to find suitable compound for use as IS. 5-FU was finally selected as the IS for the determination of NA and NUA.

### 3.3. Optimization of Sample Preparation

Prior to loading the sample for LC injection, the coextracted proteins should be removed from the prepared solution. Different extraction procedures like protein precipitation (PPT), liquid-liquid extraction (LLE), and solid phase extraction (SPE) were tested. The largely polar character of NA and NUA makes it difficult to extract from plasma with organic solvents. In this study, NA and NUA were prepared by plasma protein precipitation. Moreover, when acetonitrile was chosen for protein precipitation, the extraction recovery was higher when methanol was used. A reconstitution procedure for the dried extract employed that optimized recovery of all analytes while at the same time sufficiently reduced the presence of endogenous matrices.

### 3.4. Method Validation

#### 3.4.1. Assay Specificity

**Figure 2:**
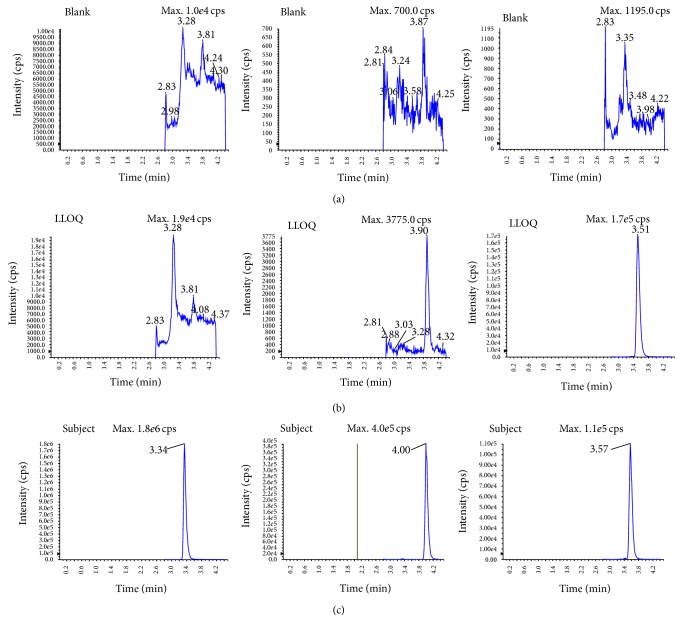
Representative MRM chromatograms for NA (I), NUA (II), and IS 5-FU (III) in human plasma: (a) blank plasma sample; (b) blank plasma sample spiked with NA and NUA at the lowest limit of quantification (5 ng/mL) and IS (1000 ng/mL); (c) plasma sample obtained from a subject 12 h after oral administration of 3 tablets of Niacin extended-release/Simvastatin.

Method specificity was demonstrated by comparing the MRM chromatograms of blank samples with those of spiked samples. No interference was detected from endogenous substances within the analytes and IS (Figure 2).

#### 3.4.2. Linearity of Calibration Curves and Lower Limit of Quantification

The linear ranges were both 5–800 ng/mL for NA and for NUA in the human plasma with correlation coefficients of >0.995. The lower limits of quantification, defined as the lowest concentration measured with ±15% accuracy and ≤15% precision, were both 5 ng/mL for NA with relative errors 6.1% and NUA with relative errors 0.7%.

#### 3.4.3. Precision and Accuracy

Assay precision and accuracy were determined by using QC samples at three concentrations in replicates (*n* = 6) by performing complete analytical runs on the same day and on three consecutive days. The within- and between-run precision and accuracy for the QC samples are summarized in Table 1.

#### 3.4.4. Extraction Recovery

The average extraction recoveries for NA were 89.7 ± 2.5%, 93.3 ± 6.3%, and 90.4 ± 5.4% at the concentrations of 10, 60, and 600 ng/mL, respectively. For NUA, the mean recoveries were 100.7 ± 7.3%, 103.0 ± 7.1%, and 98.3 ± 2.4% at the concentrations of 10, 60, and 600 ng/mL, respectively. The extraction recovery of the IS was 90.2 ± 3.7% (5-FU).

#### 3.4.5. Matrix Effect

Coeluting matrix compounds, in the plasma samples, may reduce or enhance the ion intensity of the analytes and affect the reproducibility and accuracy of the assay. The matrix effect was assessed as follows: to the blank matrix from six different individuals, analytes were added at three concentrations (low, middle, and high), making a total of 18 samples. These samples were subjected to the analytical procedure and compared with the standard working solutions.

The relative matrix effect of NA was 80.0–84.4, 83.1–96.5, and 80.8–91.2% at the concentrations of 10, 60, and 600 ng/mL, respectively. The relative matrix effect of NUA was 81.7–96.2, 85.3–99.3, and 83.5–96.6% at the concentrations of 10, 60, and 600 ng/mL, respectively. It indicated that the matrix effects had no practical effect on the quantification of NA and NUA.

#### 3.4.6. Dilution Integrity

The relative errors for QC samples of 20-fold dilution with control matrix for NA were from −3.7% to 1.2% and 11.7% to 2.4% for NUA. The results demonstrated that plasma samples could be diluted 20-fold with control matrix with no effect on the accurate quantitation of NA and NUA.

#### 3.4.7. Stability

The stability of NA and NUA in human plasma was investigated under a variety of storage and process conditions. The results of the stability studies (Tables 2 and 3) did not reveal any significant degradation under the conditions of the experiment.

### 3.5. Application of the Method

After validation, the LC-MS/MS method was used to determine NA and NUA concentrations in plasma samples after oral administration of Niacin extended-release/Simvastatin to two groups' healthy volunteers. The results were summarized in Table 4.

**Figure 3:**
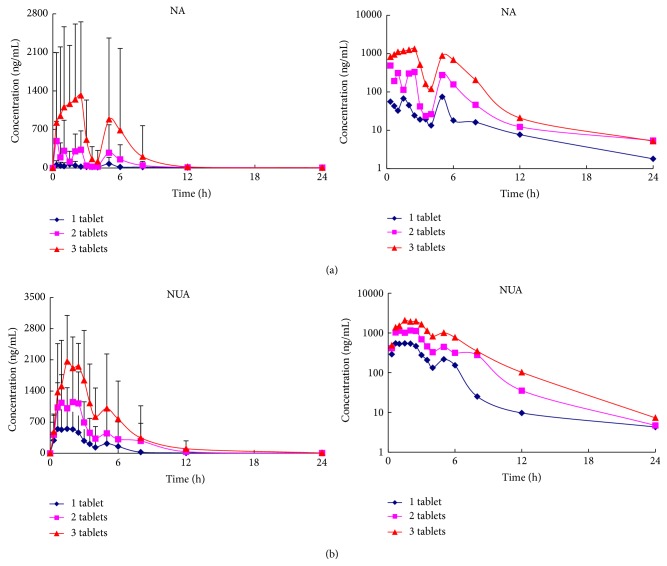
Mean plasma concentration-time profiles and logarithmic transformation profiles of NA (a) and NUA (b) after oral single dose administration of Niacin extended-release/Simvastatin (1 tablet 500 mg/10 mg, 2 tablets 1000 mg/20 mg, and 3 tablets 1500 mg/30 mg) to volunteers (*n* = 12).

Figure 3 displays the mean plasma concentration-time curve of NA and NUA. It was showed that after single oral dose of 1 tablet Niacin extended-release/Simvastatin, the mean maximum plasma concentration (*C*_max_) was 159.8 ± 125.3 and 836.0 ± 398.9 ng/mL, and the time to reach maximum concentration (*T*_max_) was 2.94 ± 2.63 and 2.06 ± 1.60 h for NA and NUA, respectively. The half-life (*t*_1/2_) was found to be 8.80 ± 8.49 and 1.70 ± 0.80 h for NA and NUA, respectively. The AUC_0−*t*_ for NA and NUA was 348.6 ± 191.7 and 2152 ± 1362 ng·h/mL, respectively. After single oral dose of 2 tablets Niacin extended-release/Simvastatin, *C*_max_ was 904.0 ± 487.0 and 1628 ± 636.0 ng/mL, and *T*_max_ was 1.82 ± 1.69 and 3.03 ± 2.19 h for NA and NUA, respectively. The *t*_1/2_ was found to be 7.33 ± 8.68 and 2.55 ± 1.26 h for NA and NUA, respectively. The AUC_0−*t*_ for NA and NUA was 1538 ± 957.0 and 5396 ± 3215 ng·h/mL, respectively. After single oral dose of 3 tablets Niacin extended-release/Simvastatin, *C*_max_ was 2476 ± 1431 and 2794 ± 1038 ng/mL, and *T*_max_ was 2.42 ± 1.64 and 2.33 ± 1.05 h for NA and NUA, respectively. The *t*_1/2_ was found to be 5.40 ± 4.57 and 2.72 ± 1.83 h for NA and NUA, respectively. The AUC_0−*t*_ for NA and NUA was 6021 ± 6579 and 10210 ± 7076 ng·h/mL, respectively.

We found that there was significant difference between subjects, while there was no significant statistical difference (*P* > 0.05) in the most main pharmacokinetic parameters of NA and NUA between treatments.

## 4. Conclusion

In this study, the LC-MS/MS method were developed and validated for the quantification of NA and its active metabolite NUA in human plasma. The method has been successfully applied to pharmacokinetic study after oral administration of a fixed dose combination tablet of Niacin extended-release/Simvastatin (500 mg/10 mg) to the humans. The results showed that the plasma concentration and pharmacokinetic performance of NA and NUA have significant difference between individuals. It indicated that much attention should be paid during the clinical use especially in coadministration when suffering from several illnesses. Our results have clinical implications and warrant further investigation of Niacin extended-release/Simvastatin.

## Figures and Tables

**Table 1:** Precision and accuracy for the analysis of NA and NUA in quality control (QC) samples (*n* = 3 days, 6 replicates per day) in human plasma.

	Concentration (ng/mL)		RSD (%)		Relative error (%)
	Added	Found	Intraday	Interday	
NA	10.0	9.72	6.4	2.8	−2.8
	60.0	61.4	8.7	9.4	2.3
	600.0	587.3	5.0	7.4	−2.2

NUA	10.0	10.3	7.6	5.8	3.2
	60.0	58.2	5.5	5.1	−2.9
	600.0	596.0	5.7	3.7	−0.6

**Table 2:** Stability data for stock solutions of NA and NUA 6 h in room temperature and 7 days at −20°C (before and after).

	Response (×10^5^)		
	Before	Six h at room temperature	Seven days at −20°C
NA	14.7	14.6	13.9
	15.5	15.6	15.4
	14.2	14.1	13.9
	14.0	15.8	14.2
	14.7	15.1	14.3
	16.9	13.8	13.1
R.E.%		−1.1	−5.8

NUA	6.0	6.8	6.1
	6.6	6.5	6.3
	6.3	6.5	6.4
	6.4	6.7	6.4
	6.5	6.3	6.6
	7.0	6.3	6.5
R.E.%		0.8	−1.6

**Table 3:** Stability data for NA and NUA in human plasma under different storage conditions (before and after analysis, *n* = 3).

	Storage conditions	Added *C*	Before *C*/R.E.	Found *C*/R.E.
		(ng/mL)	(ng/mL)/%	(ng/mL)/%
NA	Short-term (3 h at 24°C)	10.0	9.4/−5.8	9.1/−8.9
		60.0	60.2/0.4	58.8/−2.1
		600.0	611.0/2.17	636.3/6.0
	Long-term (2 months at −20°C)	10.0	9.4/−5.7	9.6/−4.1
		60.0	56.0/−6.6	53.7/−10.6
		600.0	527.0/−12.2	595.3/−0.8
	Three freeze/thaw cycles	10.0	9.4/−1.9	9.5/−4.6
		60.0	60.2/0.4	58.0/−3.28
		600.0	611.0/1.8	546.3/−9.0
	Autosampler for 4 h (24°C)	10.0	9.9/−1.0	9.6/−3.7
		60.0	61.6/2.6	62.7/4.5
		600.0	597.4/0.4	578.3/−3.6

NUA	Short-term (3 h at 24°C)	10.0	10.8/7.7	11.0/9.7
		60.0	60.1/0.2	63.6/5.9
		600.0	624.7/4.1	628.7/4.8
	Long-term (2 months at −20°C)	10.0	10.2/2.3	10.6/6.4
		60.0	60.5/0.8	59.1/−1.5
		600.0	608.0/1.3	579.3/−3.4
	Three freeze/thaw cycles	10.0	10.8/7.7	10.8/8.3
		60.0	60.1/0.2	63.3/5.6
		600.0	624.7/4.1	618.3/3.1
	Autosampler for 4 h (24°C)	10.0	10.9/9.3	10.7/7.3
		60.0	59.4/−0.9	67.0/11.7
		600.0	611.3/1.9	598.3/−0.3

**Table 4:** The main pharmacokinetic parameters for NA and NUA after single oral doses of Niacin extended-release/Simvastatin to healthy volunteers (mean ± S.D., *n* = 12).

	1 tablet		2 tablets		3 tablets	
	NA	NUA	NA	NUA	NA	NUA
AUC_0–*t*_ (*μ*g·h/mL)	0.3846 ± 0.1917	2.151 ± 1.362	1.538 ± 0.9570	5.396 ± 3.215	6.021 ± 6.579	10.21 ± 7.076
AUC_0–*∞*_ (*μ*g·h/mL)	0.5176 ± 0.2265	2.181 ± 1.368	1.719 ± 0.9230	5.478 ± 3.249	6.097 ± 6.555	10.25 ± 7.091
*t*_1/2_ (h)	8.80 ± 8.49	1.70 ± 0.801	7.33 ± 8.68	2.55 ± 1.26	5.40 ± 4.57	2.72 ± 1.83
*K*_*e*_ (1/h)	0.19 ± 0.20	0.53 ± 0.31	0.43 ± 0.69	0.33 ± 0.14	0.27 ± 0.25	0.33 ± 0.15
MRT_0–*t*_ (h)	5.37 ± 1.96	2.91 ± 0.74	4.40 ± 2.21	3.60 ± 1.32	3.65 ± 1.33	3.79 ± 1.11
*C*_max⁡_ (*μ*g/mL)	0.1598 ± 0.1253	0.8360 ± 0.3989	0.9040 ± 0.4870	1.628 ± 0.6360	2.476 ± 1.431	2.794 ± 1.038
*T*_max⁡_ (h)	2.94 ± 2.63	2.06 ± 1.60	1.82 ± 1.69	3.03 ± 2.19	2.42 ± 1.64	2.33 ± 1.05
CL_*z*_/*F* (L/h)	1271 ± 850.0	287.8 ± 128.2	744.0 ± 356.0	224.8 ± 91.90	526.0 ± 421.0	189.3 ± 94.20
*V*_*z*_/*F* (×10^3^ L)	13.71 ± 11.60	0.6490 ± 0.3380	7.649 ± 9.324	0.8210 ± 0.5230	5.199 ± 6.651	0.6960 ± 0.4580
